# Further evidence for “gain-of-function” mechanism of DFNA5 related hearing loss

**DOI:** 10.1038/s41598-018-26554-7

**Published:** 2018-05-30

**Authors:** Hongyang Wang, Jing Guan, Liping Guan, Ju Yang, Kaiwen Wu, Qiongfen Lin, Wenping Xiong, Lan Lan, Cui Zhao, Linyi Xie, Lan Yu, Dan Bing, Lidong Zhao, Dayong Wang, Qiuju Wang

**Affiliations:** 1 Chinese PLA Institute of Otolaryngology, Chinese PLA General Hospital, Medical School of Chinese PLA, Beijing, 100853 China; 2 BGI-Shenzhen, Shenzhen, 518120 China

## Abstract

To report two *DFNA5* pathogenic splice-site variations and a novel benign frameshift variation to further support the gain-of-function mechanism of *DFNA5* related hearing impairment, targeted genes capture and next generation sequencing were performed on selected members from Family 1007208, 1007081 and a sporadic case with sensorineural hearing loss. Reverse transcriptase polymerase chain reaction was conducted on the proband from Family 1007208 to test how the splice-site variation affects the transcription in RNA level. A novel heterozygous splice-site variation c.991-3 C > A in *DFNA5* was found in Family 1007208; a known hotspot heterozygous splice-site variation c.991-15_991_13delTTC was identified in Family 1007081. Both the splice-site variations were segregated with the late onset hearing loss phenotype, leading to the skipping of exon 8 at RNA level. In addition, a novel *DFNA5* frameshift variation c.116_119delAAAA was found in the sporadic case, but was not segregated with the hearing impairment phenotype. In conclusion, we identified one novel and one known pathogenic *DFNA5* splice-site variation in two Chinese Families, as well as a novel *DFNA5* frameshift variation c.116_119delAAAA in a sporadic case, which does not the cause for the hearing loss case. Both the two pathogenic splice-site variations and the nonpathogenic frameshift variation provide further support for the specific gain-of-function mechanism of *DFNA5* related hearing loss.

## Introduction

Autosomal dominant non-syndromic hearing loss (ADNSHL) accounts for approximately 20% of non-syndromic hereditary hearing loss, following by autosomal recessive non-syndromic hearing loss. To date, 37 ADNSHL related genes have been identified and more than 60 loci have been located (http://hereditaryhearingloss.org/). DFNA5 is the fifth locus of ADNSHL and the related gene is *DFNA5* (no indication for its function, so the gene was called *DFNA5* for now). The numerous ADNSHL related genes variations types include nonsense, missense, splice-site, insert and deletion (InDel) and copy number variation (CNV) and so on. For *DFNA5* (MIM #600994), only 6 splice-site variations have been reported to be pathogenic for hearing loss (Table 1)^1–8^. Although these splice-site variations are various on DNA level, all of them result in skipping of exon 8 effect on mRNA level, leading to identical gain-of-function on the protein level^9^. Except for pathogenic splice-site variations of *DFNA5*, one truncating variation in exon 5 has been reported, which did not cause hearing impairment^10^; and one nonsense variation c.781 C > T (p.R261X) in exon 7 has been identified in a Japanese family, which was also unlikely to be the disease-causing gene variation, as another pathogenic *POU4F3* variation was identified in the family affected members^11^.

**Table 1:** Overview of all DFNA5 variations identified to date.

Variation DNA	Location	Effect	Age of onset (years old)	Hearing impairment	Nationality	Reference
c.990 + 503_990 + 1691 delins132	Intron 7	Skipping of exon8	5–15	High-all frequency	Netherlands	De Leenheer *et al*., 1998
c.1183 + 4 A > G	Intron 8	Skipping of exon8	11–50	High frequency	China	Yu C *et al*., 2003
c.991–6 C > G	Intron 7	Skipping of exon8	0–40	High-all frequency	Netherlands	Bischoff A.M. *et al*., 2004
c.991–15_991–13del	Intron 7	Skipping of exon8	7–30	High frequency	China	Cheng J. *et al*., 2007
			20+	High frequency	Korea	Park H.J. *et al*., 2010
			10–30/18	High frequency	Japan	Nishio A. *et al*., 2014
			6–20	All frequency	China	This study
c.991–2 A > G	Intron 7	Skipping of exon8	8–18	High frequency	China	Chai Y. *et al*., 2014
IVS8 + 1delG	Intron8	Skipping of exon8	8–30	High-all frequency	China	Li-Yang M.N. *et al*., 2015
c.991–3 C > A	Intron7	Skipping of exon8	20–39	High-all frequency	China	This study

In this present study, by identifying a novel disease-causing *DFNA5* splice-site variation, a known pathogenic *DFNA5* splice-site variation and a novel nonpathogenic *DFNA5* truncating variation through next generation sequencing (NGS), we provide further evidence for the hypothesis that the mechanism of *DFNA5* associated hearing loss is the gain-of-function.

## Results

### Clinical description

**Figure 1:**
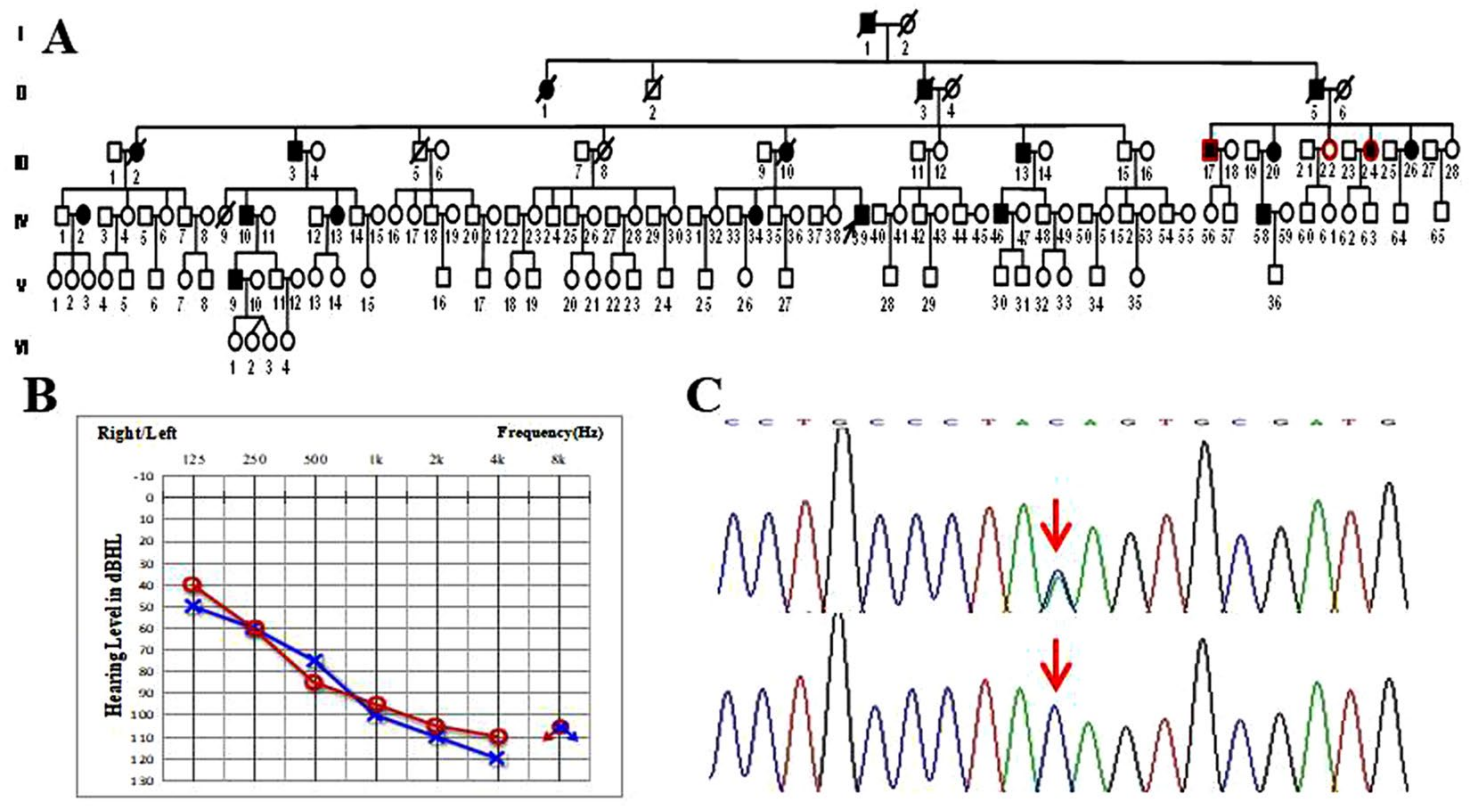
The ADNSHL Family 1007208 segregated with the *DFNA5* splice-site variation. (**A**) Pedigree of Family 1007208. Filled symbols for males (squares) and female (circles) represent affected individuals, and empty, unaffected individuals. Arrow represents the proband. Symbols with red frame indicate members whose samples were used for whole exome sequencing. (**B**) Audiogram of the proband of Family 1007208. Symbols “o” and “x” denote air conduction pure-tone thresholds at different frequencies in the right and left ear. dB, decibels; Hz, Hertz. (**C**) DNA sequence chromatograms showing the splice-site variations c.991-3 C>A in affected individuals (upper panel) compared with the wild type controls (lower panel).

For Family 1007208, a total of 22 family members, including 10 clinically affected and 12 unaffected individuals were ascertained in this study (Fig. 1). In this family, the 10 affected members showed symmetrical and bilateral non-syndromic sensorineural hearing loss, onset age of most of whom were at the third or the forth ten years. The pure-tone average (PTA) of the affected members ranged from 63.75 to 101.25 dB HL. Auditory brainstem responses (ABR) could not be evoked in both ears, and distortion product otoacoustic emissions (DPOAE) of the propositus (IV:39) were absent at all frequencies. Speech recognition in both the left and right ears of the propositus (IV:39) was 0%, with an acoustic stimulus intensity of 100 dBnHL. Some patients in this family reported accompanied tinnitus but no vestibular symptoms or signs (Table 2). The caloric tests and the amplitude of the cervical vestibular evoked myogenic potential (cVEMP) of the propositus (IV:39) were normal. High resolution computed tomography (HRCT) of the propositus (IV:39) showed normal middle and inner ear structures. No history of exposure that might account for their hearing impairment. No related systematic findings were identified through examination of medical histories or physical examination. One of the unaffected members, VI:1 (11 years old), had a variation but showed no hearing impairment affecting all frequencies at the time of hearing testing. The PTAs of VI:1 were 10 dB HL in the left ear and 12.5 dB HL in the right ear (Supplement file 2 and 3).

**Table 2:** Summary of clinical data for hearing impaired members in Family 1007208 and Family 1007081.

Family	Subject	Gender^a^	Age of test (year)	Age of onset (year)	PTA (dB HL)^b^	Hearing impairment^c^	Audiogram	Tinnitus	Vertigo
1007208	III:3	M	77	39	72.50	severe	flat	−	−
	III:17	M	59	20	77.50	severe	flat	+	−
	III:20	F	58	N/A	91.25	profound	downslope	+	−
	III:24	F	50	21	63.75	moderately severe	flat	+	−
	III:26	F	48	20	66.25	moderately severe	flat	+	−
	IV:10	M	57	29	70.00	moderately severe	flat	+	−
	IV:13	F	55	26	87.50	severe	downslope	+	−
	IV:34	F	41	N/A	101.25	profound	downslope	+	−
	IV:39	M	36	22	98.75	profound	downslope	+	−
	IV:58	M	37	21	96.25	profound	downslope	+	−
1007081	II:9	M	50	20	73.75	severe	downslope	+	−
	III:14	M	25	13	66.25	moderately severe	flat	+	−
	III:15	F	22	6	95.00	profound	flat	+	−

^a^M, male; F, female.

^b^PTA, pure-tone averages (0.5, 1, 2 and 4 kHz) for the better-hearing ear of affected subjects in Family 1007208.

^c^Diagnosed at the time of test. The severity of hearing impairment was defined as mild (26–40 dB HL), moderate (41–55 dB HL), moderately severe (56–70 dB HL), severe (71–90 dB HL) and profound (>90 dB HL).

N/A, not available; + , positive finding; −, negative finding.

**Figure 2:**
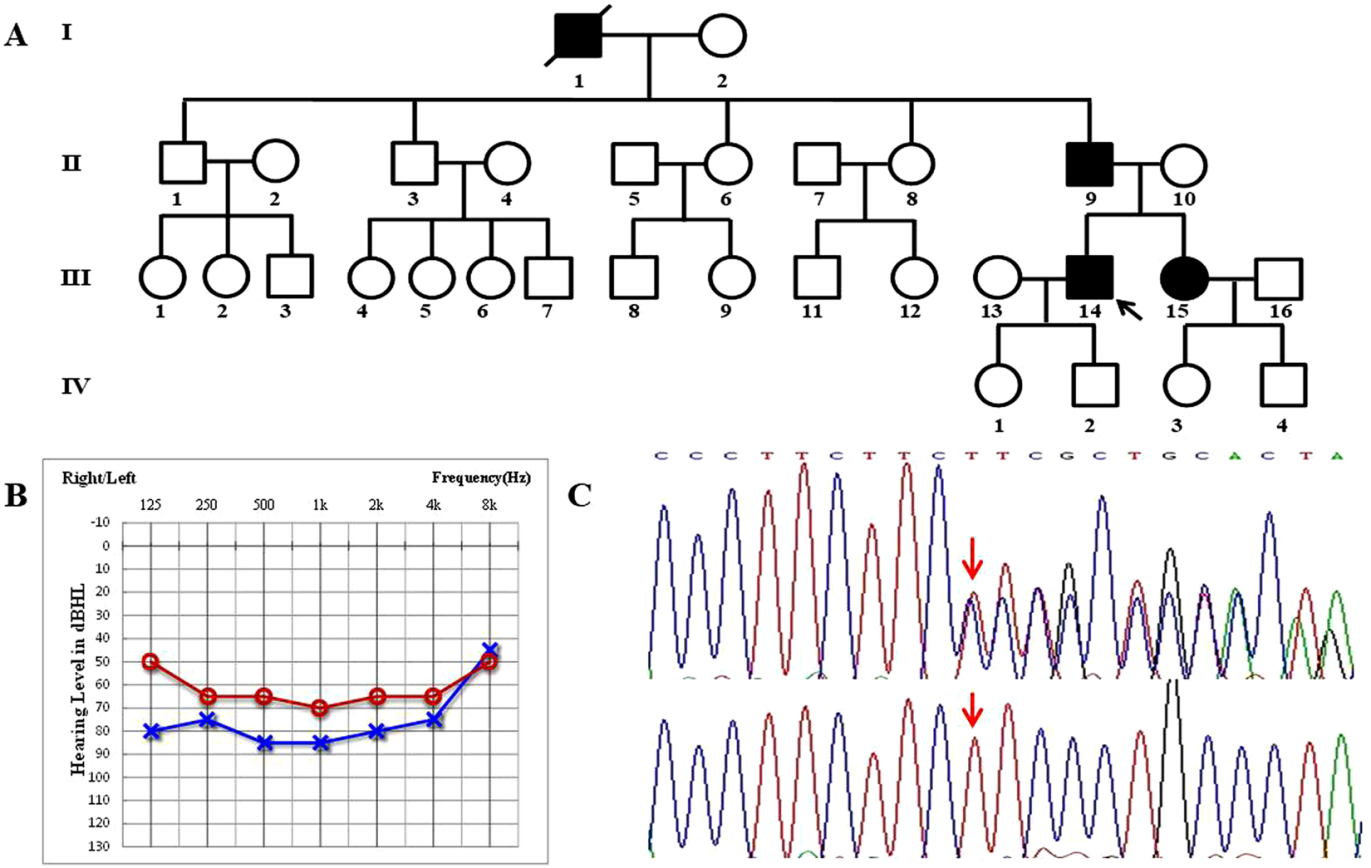
The ADNSHL Family 1007081 segregated with the *DFNA5* splice-site variation. (**A**) Pedigree of Family 1007081. Filled symbols for males (squares) and female (circles) represent affected individuals, and empty, unaffected individuals. Arrow represents the proband. Symbols with red frame indicate members whose samples were used for targeted exome sequencing. (**B**) Audiograms of the proband of Family 1007081. Symbols “o” and “x” denote air conduction pure-tone thresholds at different frequencies in the right and left ear. dB, decibels; Hz, Hertz. (**C**) DNA sequence chromatograms showing the splice-site variations c.991–15_991_13delTTC in affected individuals (upper panel) compared with the wild type controls (lower panel).

Three cases and eight normal members were ascertained from Family 1007081 (Fig. 2). All of the three affected members had late-onset progressive sensorineural bilateral hearing impairment, accompanied by tinnitus (Table 2).

**Figure 3:**
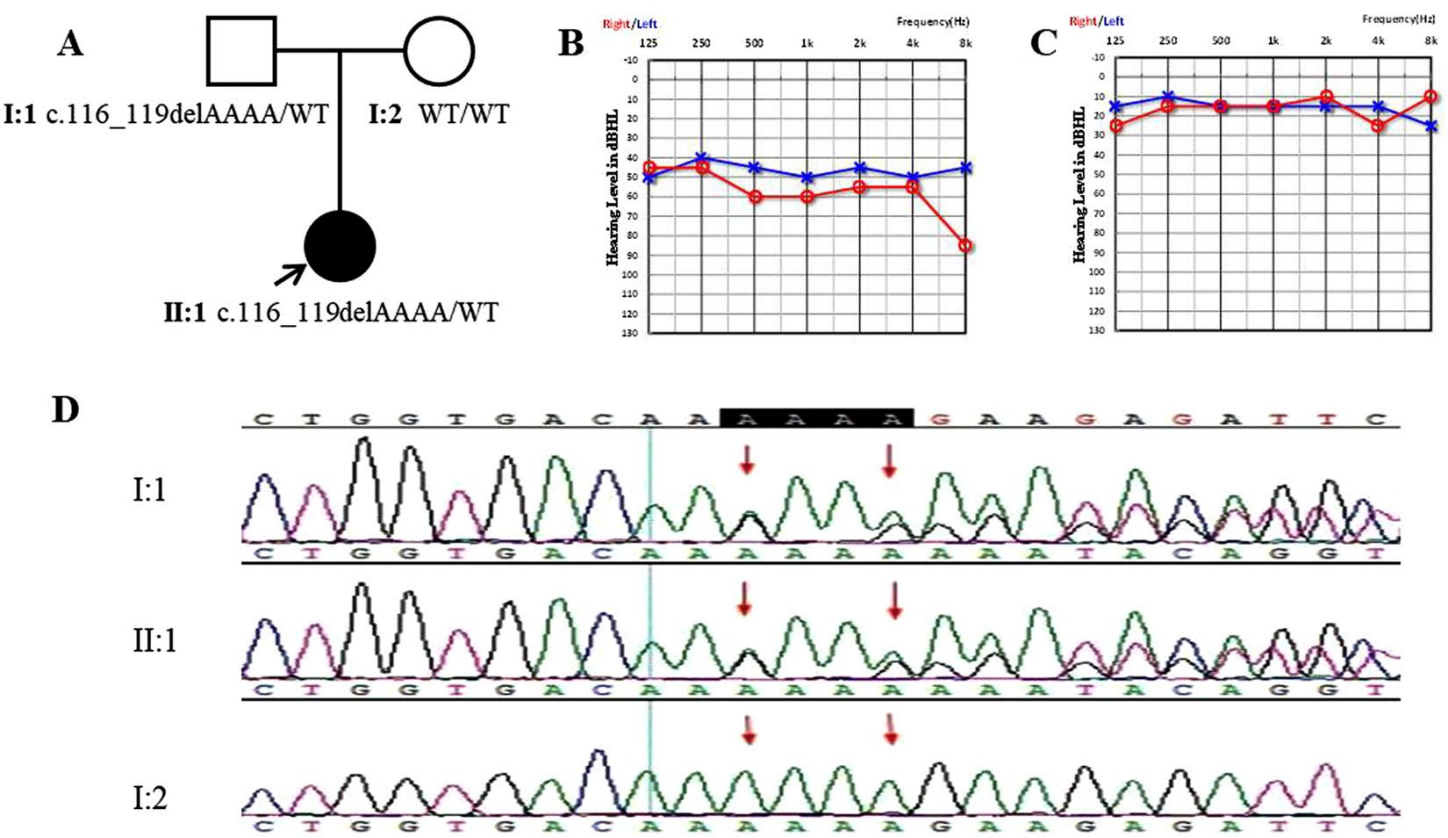
The sporadic case with the *DFNA5* frameshift variation c.116_119delAAAA. (**A**) Pedigree of the sporadic case. Arrow represents the proband. Symbols with red frame indicate members whose samples were used for targeted exome sequencing. (**B**) Audiogram of the I:1. Symbols “o” and “x” denote air conduction pure-tone thresholds at different frequencies in the right and left ear. dB, decibels; Hz, Hertz. (**C**) Audiogram of the II:1, the proband’s father, who was 38 years old when testing. (**D**) DNA sequence chromatograms showing the deletion variation c.116_119delAAAA both in the affected proband and his father, while his mother (II:2) had wild type.

For the sporadic case, who was 12 years old when she first visited our clinic, no family history was described in this core family (Fig. 3A). She had moderated hearing loss and the PTA of left and right ear were 47.5 dB HL and 57.5 dB HL, respectively. The hearing loss was presented at all frequencies (Fig. 3B). The father of this case, who also has the DFNA5 frameshift variation, showed normal hearing when tested at 38 years old (Fig. 3C). The onset age of this affected case was 11 years old.

### High-throughput sequencing results and variation analysis

For 1007208, after the filtration of common and low frequency variations, there were 51 high confidence potentially harmful variants located on the coding region which were shared by two cases but not in normal control. Combining the expression information from MGI database, we select the genes that can be detected in inner ear. Then 7 variants located on 7 genes (*MFI2/DFNA5/NSUN5/TECPR1/MLLT3/CNOT1/AKAP8*) were reserved. The same bioinformatic analysis was performed on Family 1007081 and the sporadic case.

Sanger sequencing confirmed the co-segregation of the c.991-3 C>A in *DFNA5* with the disease phenotype in Family 1007208, and the c.991-15_991_13delTTC with the phenotype in Family 1007081. The variation was not detected in the normal unaffected members of the families, whereas all of the affected members carried the variation. The novel c.991-3 C>A variation occurred at highly conserved amino acids, and the DFNA5 c.991-3 C>A variation was not identified in the 1700 control genomic DNA samples from a panel of unaffected Chinese individuals. Whole exome sequencing did not reveal any others possible disease-causing variations or modifier genes. On the basis of these results, and the phenotypes of the family, and according to the ACMG standards and guidelines, the variation identified in this study is pathogenic according up to the standards of ACMG^12^.

### Reverse transcriptase PCR

**Figure 4:**
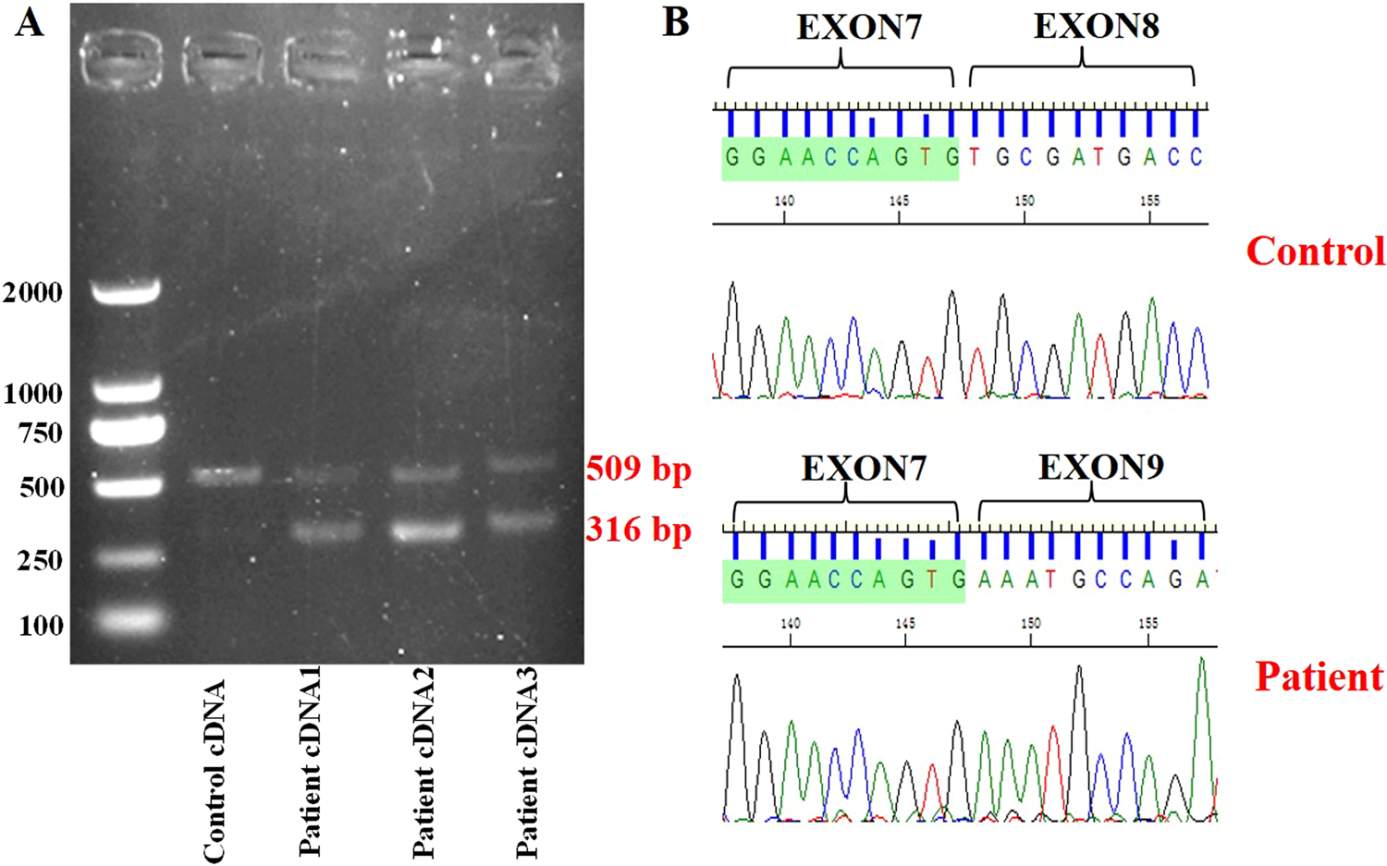
Reverse transcriptase PCR analysis of the splice-site variation. (**A**) Gel electrophoresis of cDNA products. The top band (509 bp) indicates the presence of the wildtype DFNA5 exon8, the bottom band (316 bp) represents the the exclusion of exon8. (**B**) cDNA sequence chromatograms showing the skipping of exon8 in affected patients (upper panel) compared with the wild type controls (lower panel).

A cDNA fragment of approximately 500 bp in the normal control and an additional about 300 bp fragment in the three patients of Family 1007208 were found by gel electrophoresis (Fig. 4A). The cDNA sequencing results of the 300 bp fragment of the patients showed the skipping the exon8, which has 193 bps (Fig. 4B).

## Conclusion

The hearing impairment in all of the affected members in Family 1007208 and 1007081 involved high frequency or the whole frequency. The severity of the ten affected individual in Family 1007208 and the three patients in Family 1007081 revealed no obvious tendency of the progression with aging. Like the phenotype of the family with c.991-2 A>G, the severity and the audiogram type of the affected members in this study are also variable intrafamilially^6^. The onset ages of the *DFNA5* related hearing loss ranges from 0 to 50 years old (Table 1). There is only one report of congenital hearing impairment in a Dutch family^3^, all of the other cases showed late-onset hearing loss. The onset age of the affected members in this study centralized in the third and the forth decade. Like DFNA2 (*KCNQ4* and *GJB3*), *DFNA5* is also predicted to be a candidate gene for age-related hearing loss (presbyacusis) since *DFNA5* variation related hearing loss showed similar phenotypic with presbyacusis. To investigate the association of DFNA5 with presbyacusis may provide insight into the presbyacusis mechanism. In addition, *DFNA5* was also proposed to be related to noise induced hearing loss in a case-control study^13^.

The origin of the *DFNA5* related hearing loss are concentrated in Europe and Asia, among the previous reported eleven families which have identified DFNA5 splice-site variations, only two of them originated from Europe, while all of the other nine families come from Asia (Table 1). The c.991-15_991-13del variation, identified in Family 1007081, is recurrent in China, Korea and Japan, which is a hotspot and supposed to have a founder effect and derive from a single origin by variation-linked haplotype^4,5,7^. This is the second report of this disease-causing variation in China^4^. Haplotype analysis between Chinese and Korea *DFNA5* variation related patients showed that the 3 bp deletion originated from a single origin^5^, and the haplotypes compare between Chinese and Japanese suggested that they shared region spanning 41,874 bp^7^.

To date, a total of seven variations of *DFNA5* in eleven families have been identified, all of which are splice-site variations (Table 1). All of these variations cause skipping of exon 8 and lead to premature termination of the open reading frame. It is relatively rare for the mechanism that skipping of one specific exon leads to a specific phenotype, not being caused by other variation in this gene. The hypothesis of the *DFNA5* variations gain-of-function mechanism of hearing impairment based on: 1) all of these splice-site variations, though different on DNA level, they all lead to identical effect on mRNA level and protein level in part of the studies; 2) it is not the haploinsufficiency mechanism like other ADNSHL related genes (the ratio of the normal splicing transcript to the mutant splicing transcript is more than 2:1 because the normal splicing transcript in the exon 8 is affected^2^; 3) *DFNA5* knock-out mice had no significant hearing impairment, though mutant mice showed the additional fourth row outer hair cells^14^; 4) Mutant *DFNA5* leads to cell death when transfected into mammalian cell lines (HEK293T and COS-1) and yeast cells^15^. Except for splice-site variations, no other variation types have been reported to be disease-causing for hearing impairment. In previous study, a frameshift variation c.640insC has been proved to be not pathogenic due to it was not segregated with the hearing loss phenotype^10^, the frameshift variation c.116_119delAAAA in this study is also not disease-causing according to the current evidence we got.

Since *DFNA5* was identified in a Dutch ADNSHL family in 1998^1^, numerous studies have focused on it, both relating to hearing loss and involving in cancer. DFNA5 is expressed in the cochlear hair cells and is a regulator of apoptosis cell disassembly. The molecular mechanisms of *DFNA5* related hearing loss and cancer are totally different. *DFNA5* variations, which lead to hearing loss, are expected to increase apoptosis, while for *DFNA5* involving in cancer, *DFNA5* loses its capacity to reduce apoptosis^9^. The function of DFNA5 protein is unclear, which is proposed to play a role in apoptosis and in a p53-regulated response to DNA damage. Neither mice nor zebrafishes are ideal model for clinical phenotype of *DFNA5* related hearing loss, though they have expression of the *DFNA5* homologous gene. In a patient with *DFNA5* pathogenic variation, histopathology of the inner ear showed the loss of inner and outer hair cells and severe degeneration of the stria vascularis and spiral ligament throughout the cochlea^16^. In human cell lines with mutant *DFNA5*, MAP-kinase-related activity was up-regulated, inhibition of which could partially attenuate the mutant *DFNA5* induced cell death^17^.

In conclusion, we reported a novel *DFNA5* splice-site variation c.991-3 C > A and a known recurrent *DFNA5* splice-site variation c.991-15_991_13delTTC in two independent families, which were co-segregated with the post-lingual autosomal dominant hearing impairment phenotype. No other pathogenic variations or modifier genes were identified. RT-PCR of *DFNA5* expression revealed that affected cases had less than half expression than normal control, which may play a gain-of-function role in hearing impairment. In addition, we also identified a frameshift variation in DFNA5 gene, which was not segregated in the family, further supporting the gain-of-function mechanism. However, the mechanism of the hearing loss and the specific variation-induced pathological changes *in vivo* remain unclear, even little information is available for humans, many more experiments and variations are needed to decipher their genetic code.

## Methods and Materials

### Ethics Statement

The study was approved by the Committee of Medical Ethics of Chinese PLA General Hospital. Written informed consents from all the participants in the family were obtained. The methods were performed in accordance with the approved guidelines.

### Family Recruitment and Clinical Evaluations

Two hearing loss families, including a five-generation family (Family 1007208) with 141 members and a four-generation family (Family 1007081) with 11 members segregating ADNSHL, and a sporadic case without family history were ascertained from the Chinese PLA Institute of Otolaryngology, Chinese PLA General Hospital (Figs 1, 2, 3). Personal or family medical evidence of hearing loss, tinnitus, vestibular symptoms and other clinical abnormalities of both the affected members and the unaffected members in these families were identified. Audiometric evaluations included audiogram, ABR, DPOAE, and speech recognition score. For the ABR testing, the stimulation sound was alternating short sound, with the maximum stimulus intensity of 100 dBnHL, the stimulation repetition rate is 19.3 times per second, and the number of superposition is 1024 times. PTA and the definition of severity of hearing impairment were referenced previous studies^18^. Caloric testing was performed on some patients to obtain data on semicircular canal function, and cVEMP was tested with tone burst stimulus type (500 Hz) and 100 dBnHL stimulus intensity. Some patients who had tinnitus were estimated by tinnitus assessment. HRCT was performed on the proband of Family 1007208 to verify whether the family members had other complications other than hearing disorders.

A cohort of 1700 ethnically matched normal individuals comprised the control genomic DNA samples group.

### Whole exome sequencing and targeted genes sequencing

Genomic DNA was extracted from the whole blood samples using the Blood DNA kit according to the standard protocol (Tiangen Biotech, Beijing, China). Whole exome sequencing and target region sequencing were performed on these three pedigrees. Using Nimblegen SeqCapEZ Exome v3.0 kit and Illumina HiSeq. 2000 platform, we obtained the whole exome sequencing of sample II-1, II-3 and II-4 from family 1007208. II-1 and II-4 are cases. II-3 is a normal control. Coverage of exome region was 99.10% and mean depth was 102.95X. The other pedigrees 1007081 and the sporadic case used target region capture panel containing 127 hearing loss known causative genes and Illumina HiSeq. 4000 platform, which were conducted according to the previous procedures^17^.

After sequencing and quality assessment, we aligned the reads onto the human reference genome hg19 (NCBI build 37.1) by software BWA (version 0.7.10) and SOAP aligner/SOAP2 (version 2.21). Then we use GATK (version 3.3–0), Samtools (version 0.1.19), SOAPsnp (version 1.03) and Platypus (version 0.7.2) to indentify SNPs and Indels. To obtain rare variations, common variants and low frequency variants in the 1000 Human Genomes Project database, HapMap Project database, ExAC database, EVS database and BGI in-house databases were excluded (0.5%). All remaining variants were considered to be rare variations and were annotated using Ensembl VEP, OMIM, MGI, Gene Ontology, HGNC gene annotation database and so on. Then we reserved harmful variations located on coding region including missense, nonsense, frameshift, indel and splice-site variations (Supplement file 1). Candidate variants were validated by Sanger sequencing. Variation interpretation (evaluation of the pathogenicity) was based on the standards and guidelines of the American College of Medical Genetics and Genomics and the Association for Molecular Pathology (ACMG and AMP)^12^.

### Sanger sequencing

PCR and Sanger sequencing was performed on all 22 available members from Family 1007208 and all 11 persons from Family 1007081 to determine whether the potential variations in causative genes segregated with the disease phenotype in these families or not. Direct PCR products were sequenced using Bigdye terminator v3.1 cycle sequencing kits (Applied Biosystems. Foster City, CA) and analyzed using an ABI 3700XL Genetic Analyzer (primers for c.991-3 C>A and c.991-15_991_13delTTC were DFNA5-forward: ACTTGTTTCTGGTCCCTGGC and DFNA5-reverse: TCCCACCTCTTACCCTCCCT; primers for c.116_119delAAAA were DFNA5-forward: AGGGATTGTTTCTGAAGCGT and DFNA5-reverse: GGATCATCCTTTCCGAGACTAA).

### Reverse transcriptase PCR

Total RNA from three patients and one normal control of Family 1007208 were extracted from the white cells of the whole blood samples using the cell RAN kit according to the standard protocol (Trizol reagent, Invitrogen,Carlsbad, CA), and RNA concentration and purity were determined on a NanoDrop spectrophotometer. Synthesis of cDNA was carried out following the Revert Aid First Strand cDNA Synthesis Kit (Thermo Scientific). RT-PCR was performed by using Takara SYBR Premix Ex Taq (Tli RNaseH Plus), the two alleles were amplified separately three times using the same quantity of the same template. TaqMan 7700 Sequence Detection System was used to monitor in real time the increase in fluorescent signal. Primers for cDNA sequencing were: forward primer: 5′- GCTGCGCATGGGATATCTTC -3′; reverse primer: 5′- TTCAGGGGAGTCAAGGTTGG -3′ (product size: 509 bp).

## Electronic supplementary material

Supplementary file
