# Supplementary material for: Further evidence for “gain-of-function” mechanism of DFNA5 related hearing loss
Source: Sci Rep. 2018 May 30;8:8424. doi: 10.1038/s41598-018-26554-7 (PMC5976723; doi:10.1038/s41598-018-26554-7)
Supplement: Supplementary file 1 — Supplementary file [file 41598_2018_26554_MOESM1_ESM.pdf]

Further evidence for "gain-of-function" mechanism of DFNA5 related hearing loss

Hongyang Wang[1,△], Jing Guan[1,△], Liping Guan[2,△], Ju Yang[1], Kaiwen Wu[1], Qiongfen Lin[2], Wenping Xiong[1], Lan Lan[1], Cui Zhao[1], Linyi Xie[1], Lan Yu[1], Dan Bing[1], Lidong Zhao[1], Dayong Wang[1], Qiuju Wang[1,*]

[1]Chinese PLA Institute of Otolaryngology, Chinese PLA General Hospital, Medical School of Chinese PLA, Beijing, 100853, China.
[2]BGI-Shenzhen, Shenzhen, 518120, China

Email: * wqcr301@vip.sina.com
[△]These authors contributed equally to this work.

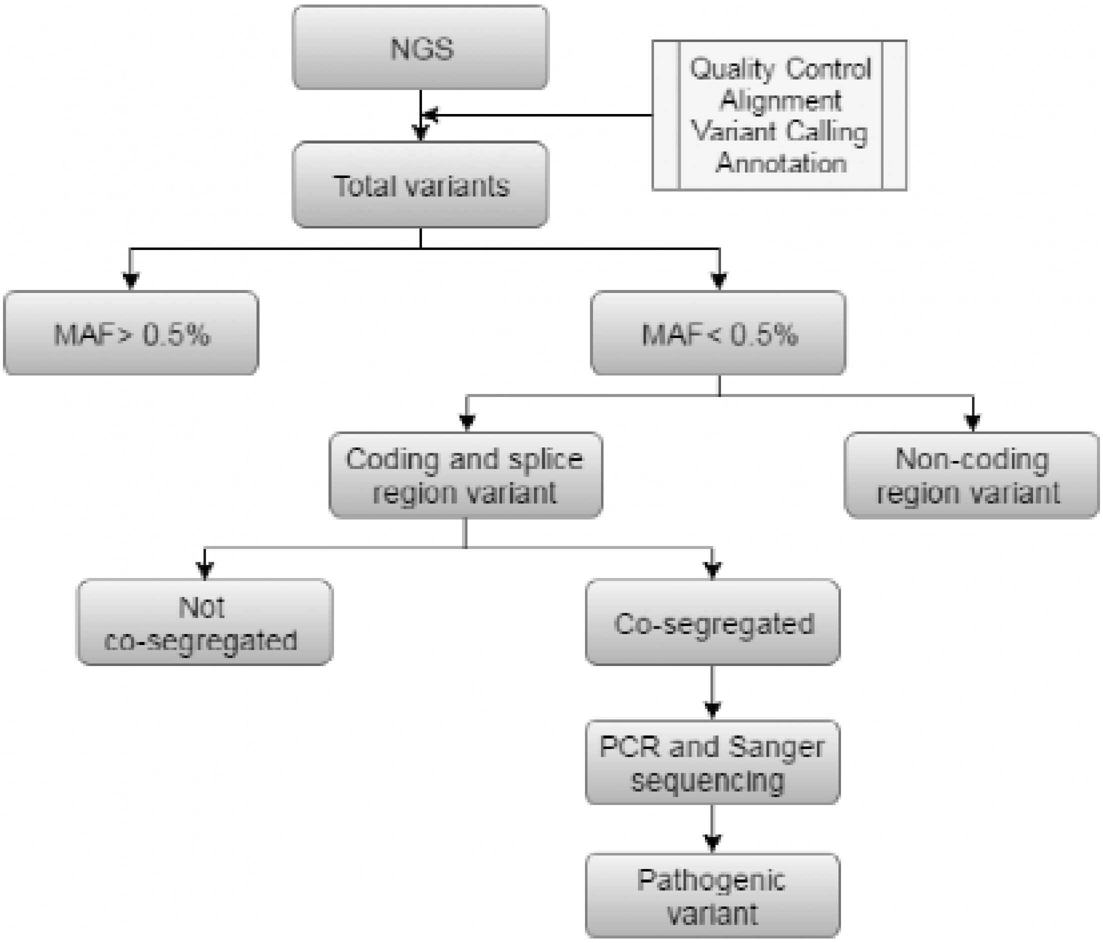

Supplement file 1. The flowchart of bioinformation analysis. After filteration of public frequency databases and using the phenotype information of every pedigree, these rare mutations that were carried by cases but none in controls were reserved. Then extend each pedigree with none-NGS samples to confirm the possibly causative variants.

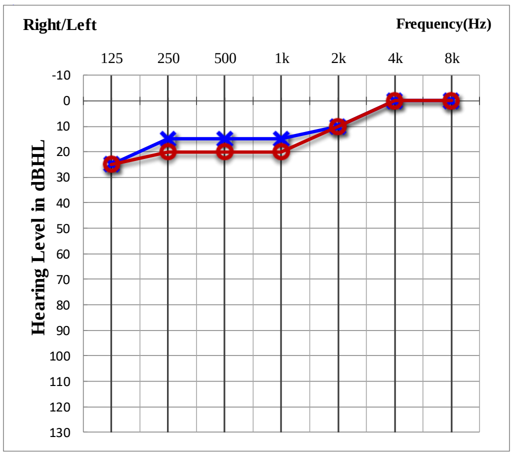

Supplement file 2. The audiogram of the VI:1 in Family 1007208.

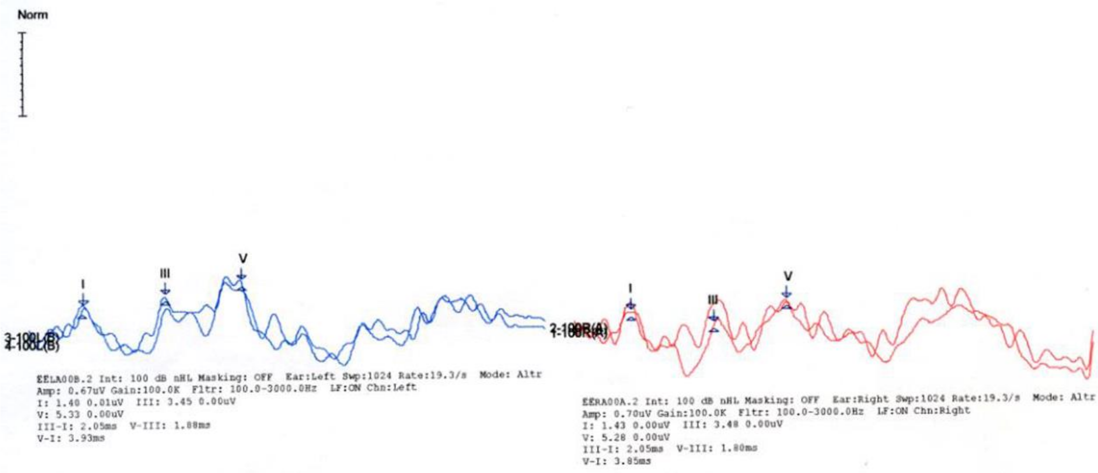

Supplement file 3. The ABR results of the VI:1 in Family 1007208.
